# The use of a bipolar single-needle electrode for electrochemotherapy combined with gene electrotransfer of IL-12 in a mast cell tumor on the nasal planum of a dog: case report

**DOI:** 10.3389/fvets.2026.1813360

**Published:** 2026-04-29

**Authors:** Ursa Lampreht Tratar, Tanja Svara, Maja Cemazar, Gregor Sersa, Natasa Tozon Mask

**Affiliations:** 1 Institute of Oncology Ljubljana, Ljubljana, Slovenia; 2 Veterinary Faculty, University of Ljubljana, Ljubljana, Slovenia; 3 Faculty of Medicine, University of Ljubljana, Ljubljana, Slovenia; 4 Faculty of Health Sciences, University of Primorska, Izola, Slovenia; 5 Faculty of Health Sciences, University of Ljubljana, Ljubljana, Slovenia

**Keywords:** dog, electrochemotherapy, gene electrotransfer, interleukin-12, single needle electrode

## Abstract

Electrochemotherapy (ECT) combined with gene electrotransfer of interleukin-12 (IL-12 GET) has shown promising results in the treatment of canine mast cell tumors (MCTs). However, when tumors are located in anatomically challenging sites, there is a need for the use of a single-needle electrode to access the tumor. Therefore, this case report aimed to evaluate the feasibility and effectiveness of using a bipolar single-needle electrode in a dog with an MCT on the nasal planum. The 2-cm^3^ tumor was treated with the simultaneous intratumoral administration of cisplatin (CDDP) and a plasmid encoding canine IL-12, followed by the application of electric pulses using a bipolar single-needle electrode. In addition, the dog was treated with a tyrosine kinase inhibitor. We observed a reduction in microvessel density 6 months after treatment, along with a decreased number of proliferating cells. These combined interventions resulted in long-term local tumor control for 18 months before recurrence occurred. Importantly, our results demonstrated that the bipolar single-needle electrode approach was both feasible and effective. In conclusion, using IL-12 GET with a bipolar single-needle electrode for the treatment of MCTs is a practical and effective therapeutic option, particularly for tumors located in difficult-to-treat anatomical sites.

## Introduction

Mast cell tumors (MCTs) are among the most common skin tumors in dogs, accounting for approximately 11 to 21% of all cutaneous neoplasms (1, 2). These tumors exhibit a wide range of biological behavior, from well-differentiated, slow-growing tumors with excellent prognosis to aggressive, high-grade lesions that rapidly progress and have a high risk of local recurrence and metastasis (3). Breed predisposition, such as in Boxers and Labradors, and tumor location significantly influence the prognosis. Specifically, MCTs on the nasal planum are rare, with only a few cases reported in the literature; therefore, the prognosis remains uncertain (4, 5). However, MCTs that arise from mucosal surfaces or mucocutaneous junctions, such as the nasal planum, are associated with a higher incidence of lymph node metastasis at the time of diagnosis (6) and exhibit more aggressive behavior, including local infiltration (7).

Electrochemotherapy (ECT) is a treatment modality that combines the use of cytotoxic drugs with electric pulses (known as electroporation) targeted directly at the tumor, which increases drug uptake and efficacy (8, 9). Drugs such as bleomycin and cisplatin are commonly used in treatment. Bleomycin works by inducing DNA breaks, while cisplatin inhibits cell repair mechanisms through the formation of DNA adducts (10, 11). ECT has been used extensively in veterinary oncology, and its efficacy has been demonstrated in the treatment of various tumor types, including MCTs, with objective response rates ranging from 75 to 95% (12–14). The therapeutic potential of ECT is further enhanced when combined with gene electrotransfer (GET) of immunomodulatory cytokines, such as interleukin-12 (IL-12) (15, 16). IL-12 GET uses electroporation to introduce plasmid DNA encoding IL-12 directly into tumor tissue or peritumoral regions. This approach stimulates the local production of IL-12 and enhances the recruitment and activation of cytotoxic T cells and natural killer cells (17). Clinical studies in veterinary oncology have confirmed that the combination of ECT and IL-12 GET leads to better local control and even systemic antitumor effects (18–21). These results support the exploration of ECT combined with IL-12 GET as a promising approach for the treatment of therapeutically challenging tumors, such as MCTs on the nasal planum. However, tumors that extend deeper into the nasal cavity in dogs remain difficult to treat due to limited accessibility. In this context, the development of a bipolar single-needle electrode represents a significant technical advancement in electroporation-based therapies. Merola et al. described the design of the bipolar single-needle electrode, which integrates both the anode and cathode on a single shaft, simplifying electrode placement and ensuring an even distribution of the electric field (22). In the preclinical setting, they showed that this design reduced invasiveness and enabled precise targeting of deep-seated tumors in complex anatomical locations, such as the nasal cavity. The electrode effectively generated irreversible electroporated volumes of tissue or tumor up to 10 mm in diameter; by performing multiple insertions, the treatment area can be extended to cover a larger region. These features make the bipolar electrode particularly suitable for electroporation-based treatment of deep-seated tumors, such as MCTs on the nasal planum (22).

This case report describes the feasibility and efficacy of using a bipolar single-needle electrode in combination with ECT and IL-12 GET to treat an MCT located on the nasal planum of a dog. This treatment approach has not been previously reported.

## Case history

**Figure 1:**
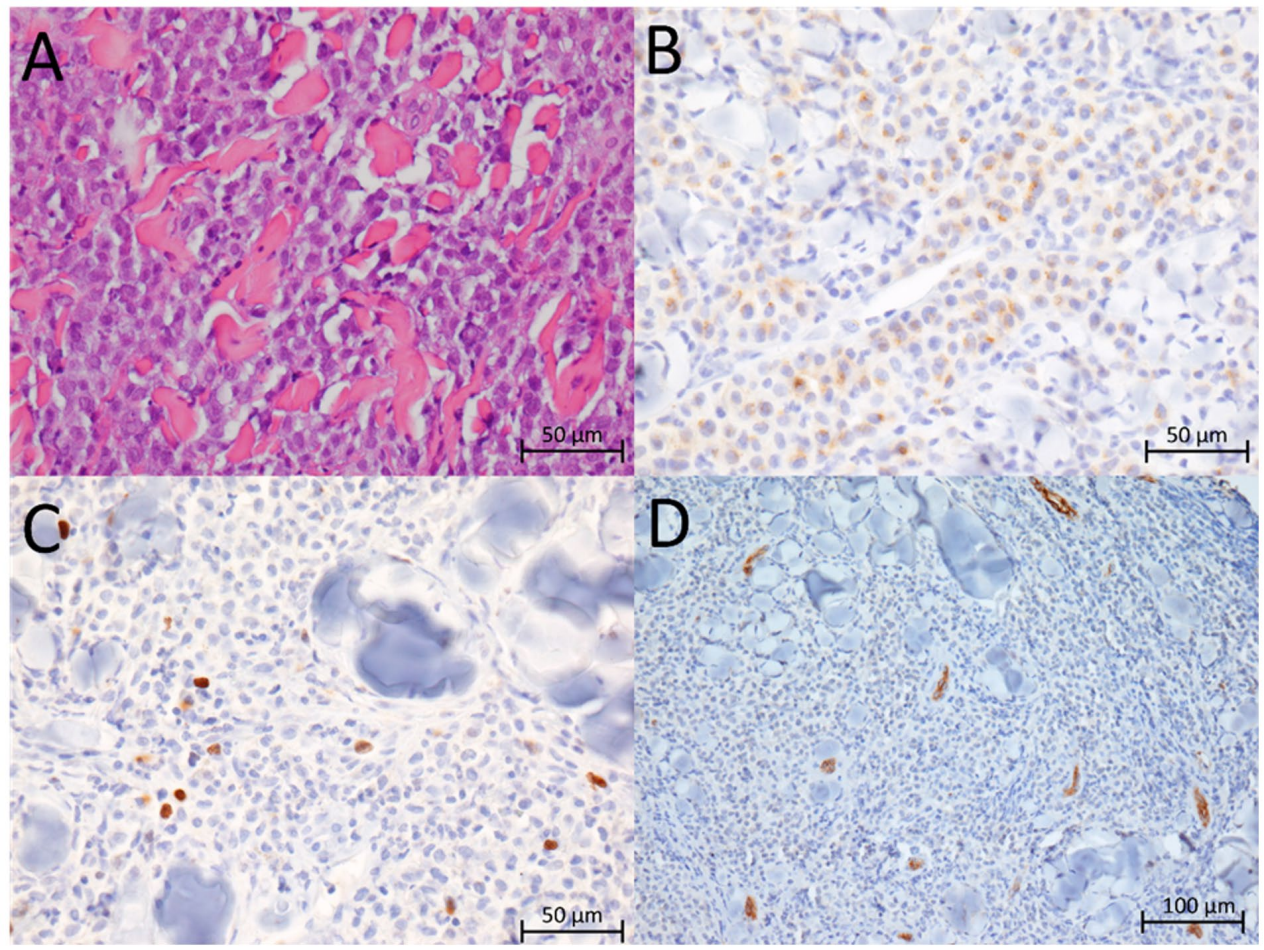
Microscopic findings in the mast cell tumor sampled before treatment: sheets of tumor cells, hematoxylin and eosin, 400× **(A)**; tumor cells showing KIT-staining pattern II, c-kit immunolabeling, 400× **(B)**; low proliferation index of tumor cells, Ki-67 immunolabeling, 400× **(C)**; representative area of the tumor with immunolabeled microvessels, von Willebrand factor/factor VIII complex immunolabeling, 200× **(D)**.

A 2-year-old, intact male Dutch Shepherd dog (Schapendoes), weighing 35 kg, presented with a mass involving the right nostril of the nasal planum. A tissue biopsy was performed at the referring veterinary clinic in June 2023. The sample was fixed in 10% buffered formalin and routinely embedded in paraffin. The tissue sections, measuring 4 μm thick, were deparaffinized, stained with hematoxylin and eosin (HE), and examined under a light microscope. Microscopic examination revealed a diagnosis of MCT, classified as Patnaik grade 2/Kiupel low grade (G2/LG). No mitosis was observed in 10 high-power fields (HPFs) (2.37 mm^2^), and there was no evidence of invasion into blood or lymphatic vessels (Figure 1A). Additional immunohistochemical staining was performed. The slides were deparaffinized, and antigens were demasked by boiling in EDTA buffer (pH 9.0) for 10 min in a microwave oven (for Ki-67 immunolabeling) or in citrate buffer (pH 6.0) for 20 min (for c-kit, von Willebrand factor/factor VIII complex, CD3, and CD20 immunolabeling). The slides were then incubated for 1 h at room temperature in a humidified chamber with the following antibodies: Monoclonal mouse anti-human Ki-67 (antigen clone MIB-1; DAKO, Glostrup, Denmark, Cat. no. M724029) diluted 1:75, rabbit polyclonal anti-human c-kit (CD117; DAKO, Cat. no. A450229) diluted 1:300, rabbit polyclonal anti-human von Willebrand factor/factor VIII complex (DAKO, Cat. no. A0082) diluted 1:50, rabbit polyclonal anti-human CD3 (DAKO, Cat. no. PAb A0452) diluted 1:100, and rabbit polyclonal anti-human CD20 (Invitrogen, Cat. no. PA5-16701) diluted 1:400. The remaining immunohistochemical procedure included blocking endogenous peroxidase activity using Dako REAL™ peroxidase blocking solution, (DAKO, code number S202386-2) for 30 min at room temperature. This blocking was performed according to a previously described protocol by Cociancich et al. (23). The immunohistochemical reaction for c-kit was evaluated according to the criteria described by Kiupel et al., and the Ki-67 proliferation index was determined as described by Šimundić et al. (24, 25). Microvessel density was determined in six random fields under 20× magnification, while the number of CD3+ and CD20+ cells was determined in six random HPFs (1.42 mm^2^). Blood vessels and CD3+ and CD20+ cells were counted using the software program NIS-Elements Basic Research (Nikon, Japan).

Focal to stippled cytoplasmic staining with decreased membrane-associated staining, corresponding to the KIT-staining pattern II, was identified in slides stained with the antibody against c-kit (Figure 1B). The proliferation index determined by Ki-67 immunostaining was low, with 5% of tumor cells showing nuclear immunolabeling (Figure 1C). The mean microvessel density before treatment was 7.2 ± 1.17 per 20× field (Figure 1D), while the mean counts of CD3+ and CD20+ cells were 3.67 ± 1.37 and 6.33 ± 7.73 per HPF, respectively.

In addition, CT imaging revealed a poorly marginated nodular lesion in the right nostril of the nasal planum, measuring 1.5 × 2.5 cm, which is isodense to the surrounding normal tissue with mild peripheral contrast enhancement. Lymphography after peritumoral injection of iodinated contrast medium showed that the sentinel lymph nodes were the right mandibular lymph nodes. Furthermore, the CT scans showed no other abnormalities. The hematological and biochemical parameters were within the normal range.

As the owners declined other treatments, including standard surgical treatment and irradiation, the dog was started on masitinib (tyrosine kinase inhibitor, Masivet^®^, 300 mg/day, AB Science, Paris, France) and prednisolone (Prednicortone^®^, 5 mg/day, Dechra, Northwich, UK) based on the results of c-kit staining. Treatment began at the end of June 2023 and continued until the end of October 2023, for a total duration of 4 months.

## Treatment course

In July 2023, the patient presented to our clinic for ECT and GET, as well as for surgical removal of the sentinel right mandibular lymph node. A fine-needle aspiration biopsy (FNAB) of the liver and spleen was performed, which showed no evidence of tumor cells. In addition, CT imaging revealed no abnormalities. The surgical resection of two mandibular lymph nodes was performed. The first lymph node measured 2 × 1 × 1 cm, and the second had a diameter of 0.8 cm. The samples were processed in the same way as the pre-treatment biopsy, with the addition of toluidine blue staining of the sections. The microscopic examination of the lymph nodes revealed reactive hyperplasia and three groups of up to 14 mast cells per HPF with mildly to moderately granulated cytoplasm were observed in the cortical parenchyma of the larger lymph node. This finding was interpreted as a pre-metastatic lymph node (HN1) according to Weishaar et al. (26).

**Figure 2:**
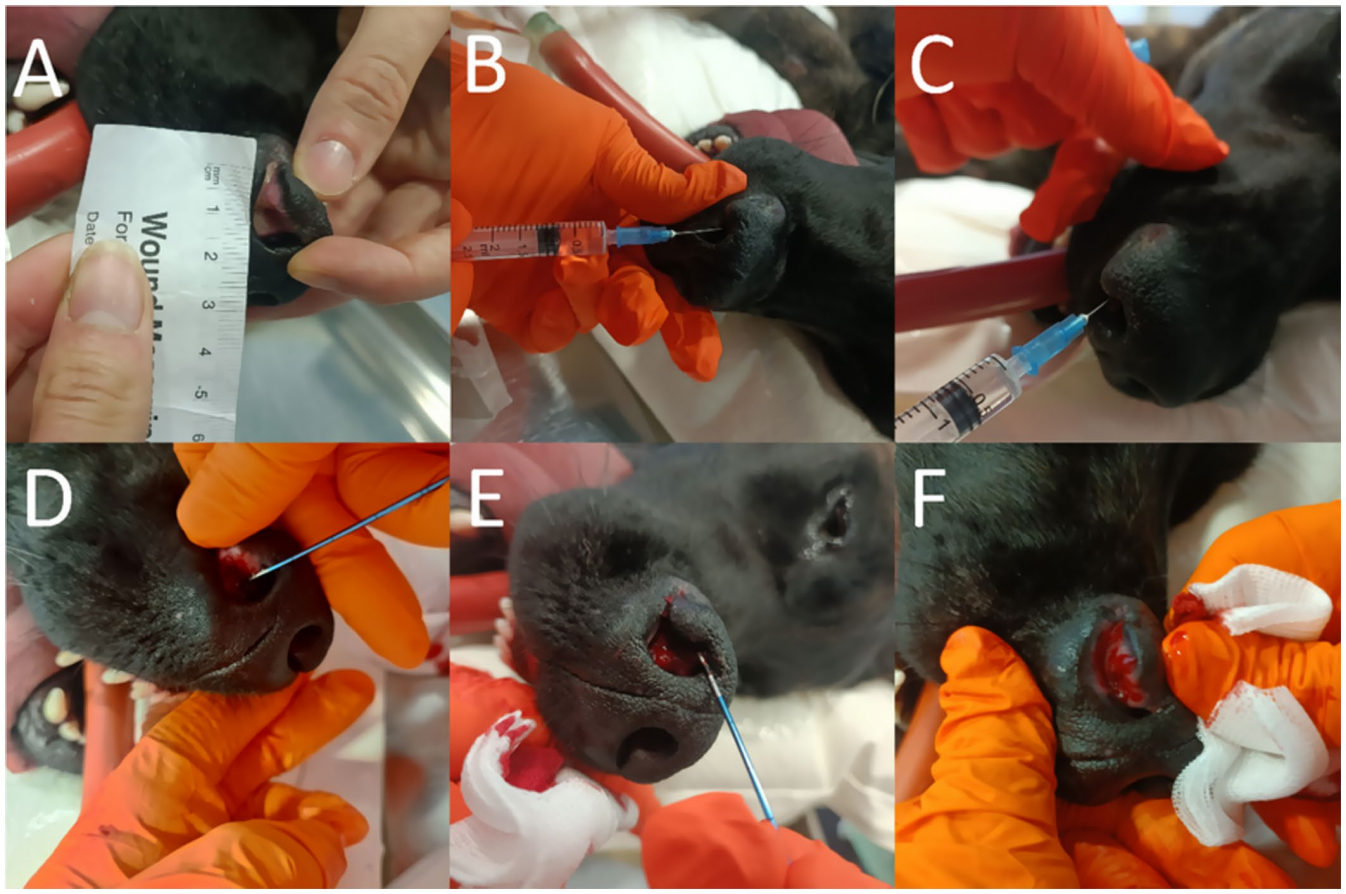
Overview of the treatment course: measurement of the tumor **(A)**, intratumoral injection of plasmid DNA **(B)**, administration of the cytostatic drug **(C)**, insertion of the bipolar electrode **(D)**, application of electric pulses **(E)**, and appearance after treatment **(F)**.

Subsequently, ECT and IL-12 GET were performed as previously described (19). The 2-cm^3^ tumor (2 × 1.6 × 1.2 cm) was first injected intratumorally with 2 mg of plasmid DNA pORFcaIL-12-ORT encoding canine IL-12 [(19, 27); Figures 2A,B]. Then, 4 min later, the tumor was injected with 1 mg of cisplatin in 1 mL of physiological saline (CDDP, cisplatin, Fresenius Kabi Austria GmbH, Graz, Avstrija Figure 2C). Furthermore, 1 min after the CDDP injection and 5 min after the plasmid injection, commercially available bipolar single-needle electrodes with a length of 16 cm, an active part of 1.3 cm, and a diameter of 1.2 mm (17G) (B-TS-SA, Igea S.p.A., Italy) were inserted into the tumor, and a series of eight short-wave pulses (100 μs) with an amplitude of 1,000 V/cm and a frequency of 5 kHz was applied (Cliniporator, Igea S.p.A., Italy, Figures 2D–F). During the treatment, the bipolar electrode was repositioned nine times to achieve complete coverage of the tumor with the electric field. Although this is a single-needle electrode and repositioning would not normally be required, we repositioned the electrode due to the size of the tumor. Therefore, the repositioning was performed as described for MCT. Briefly, the electrode was first positioned at the outer margins of the tumor and then at its center. Therefore, the degranulation that occurs during manipulation of the MCT is locally limited by the vascular lock effect (28).

## Follow-up visits

**Figure 3:**
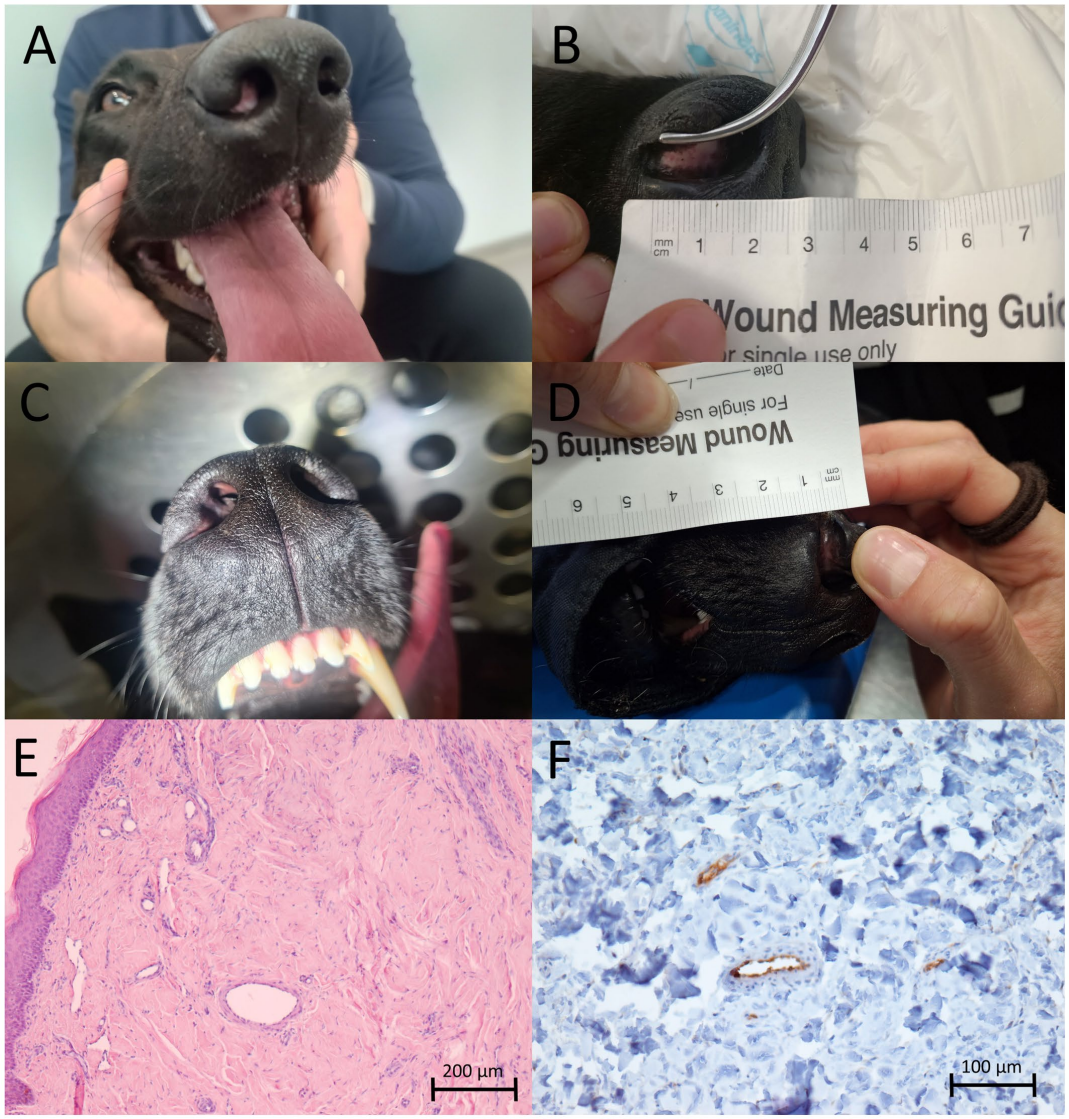
Follow-up examinations at 1 week **(A)**, 2 months **(B)**, 6 months **(C)**, and 12 months **(D)** after treatment. Microscopic findings from a biopsy taken at the tumor site 6 months after treatment: the tumor had been replaced by mature connective tissue, hematoxylin and eosin, 100× **(E)**; a representative area of the tumor showing immunolabeled microvessels, indicated by von Willebrand factor/factor VIII complex immunolabeling, 200× **(F)**.

After treatment, regular follow-up visits were scheduled at 1 week, 4 weeks, 2 months, 3 months, 6 months, 12 months, and 18 months (Figures 3A–D). A photograph was taken at each visit, and the patient was additionally anesthetized because the examination could not be performed while the dog was awake.

**Figure 4:**
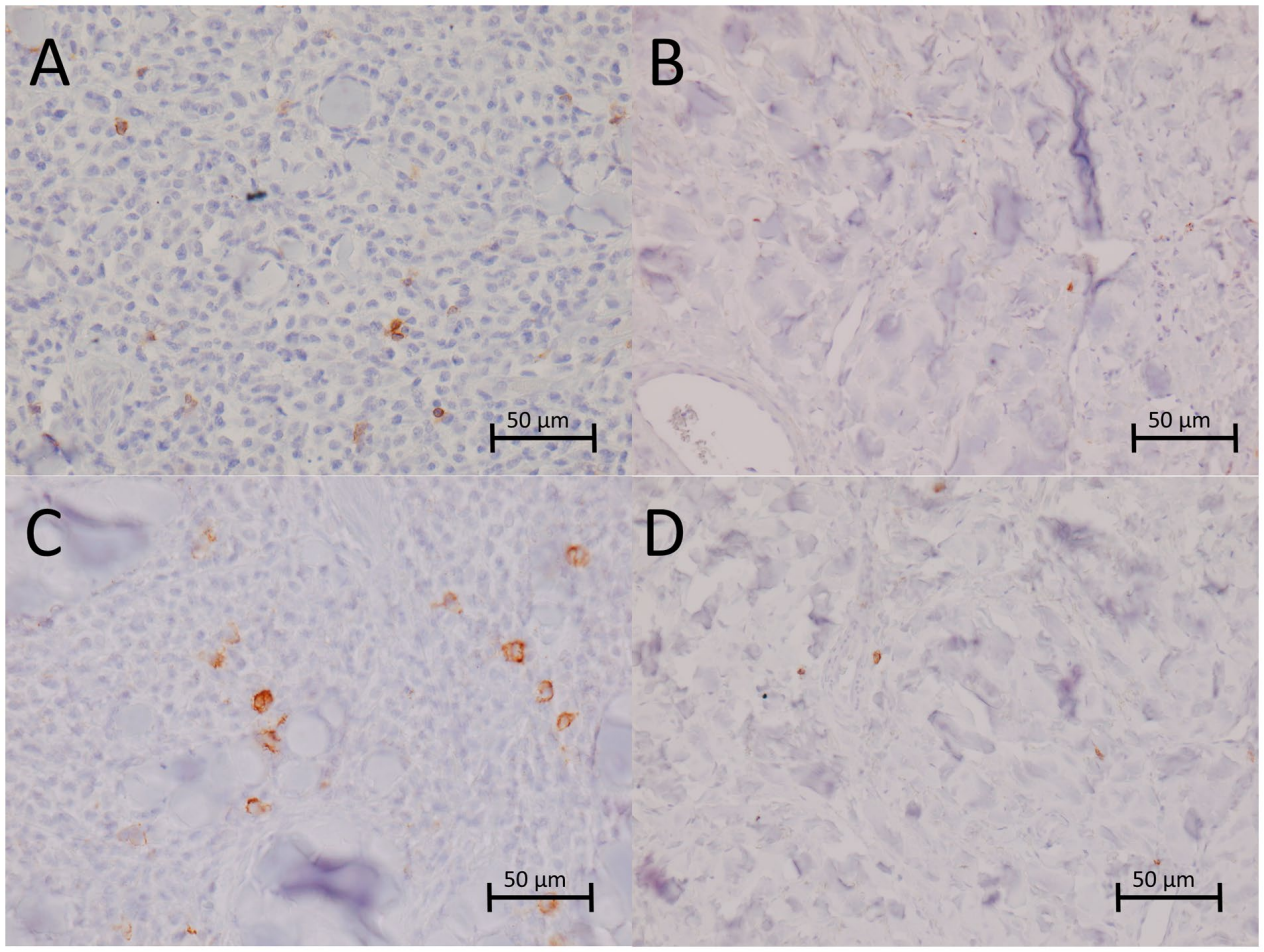
Immunohistochemical staining of CD3^+^ and CD20^+^ cells in the mast cell tumor sampled before treatment and 6 months after treatment. CD3^+^ lymphocytes infiltrating tumor tissue before treatment **(A)** and 6 months after treatment **(B)**. CD20^+^ lymphocytes infiltrating tumor tissue before treatment **(C)** and 6 months after treatment **(D)**. Hematoxylin counterstaining, 400×.

One week after treatment, edema was observed (Figure 3A), but it had resolved by 1 month post-treatment. During the 2-month follow-up visit, the dog was sedated for a thorough examination of the nasal cavity. The tumor measured 1.2 × 0.8 × 0.8 cm, representing a partial response according to the Response Evaluation Criteria in Solid Tumors in dogs (v1.0), a Veterinary Cooperative Oncology Group (VCOG) consensus document (29). At the subsequent visit 3 months after treatment, the tumor could not be measured due to the animal’s lack of cooperation; however, no significant change in size was detected. Six months after treatment, the dog underwent dental procedure and was anesthetized, allowing a second biopsy to be taken from the tumor site. Microscopic examination revealed that the tumor had been replaced by mature connective tissue, with very rare, predominantly perivascular mast cells showing no atypia (Figure 3E). However, the microscopic examination could not reliably determine whether the cells were normal or tumor mast cells. The overlying epidermis was only minimally hyperplastic. In addition, the mean microvessel density was reduced (4.0 ± 1.22 per 20 × field) (Figure 3F), as were the numbers of CD3+ cells (0.17 ± 0.41 per HPF) and CD20+ cells (0.67 ± 0.82 per HPF) compared to pre-treatment values. The reductions in microvessel density and the number of CD3+ cells were statistically significant (*p* < 0.05; Table 1; Figure 4).

**Table 1:** Histological and immunohistochemical staining results and analysis.

Parameter	Before treatment	6 months after treatment	*p*
Microvessel density (per 20× field)	7.2 ± 1.17	4.0 ± 1.22	0.004
Number of CD3+ cells per HPF	3.67 ± 1.37	0.17 ± 0.41	0.0018
Number of CD20+ cells per HPF	6.33 ± 7.37	0.67 ± 0.82	0.1186

At the follow-up visit 12 months after treatment, no visible tumor was detected. A third biopsy was obtained from the previous tumor site, and the results showed that the epidermis was normal, but there were numerous sampling artifacts in the section that made interpretation almost impossible; therefore, immunolabeling was not performed. A small number of poorly preserved mast cells were present in the section stained with toluidine blue, but an accurate assessment of their morphology was not possible.

**Figure 5:**
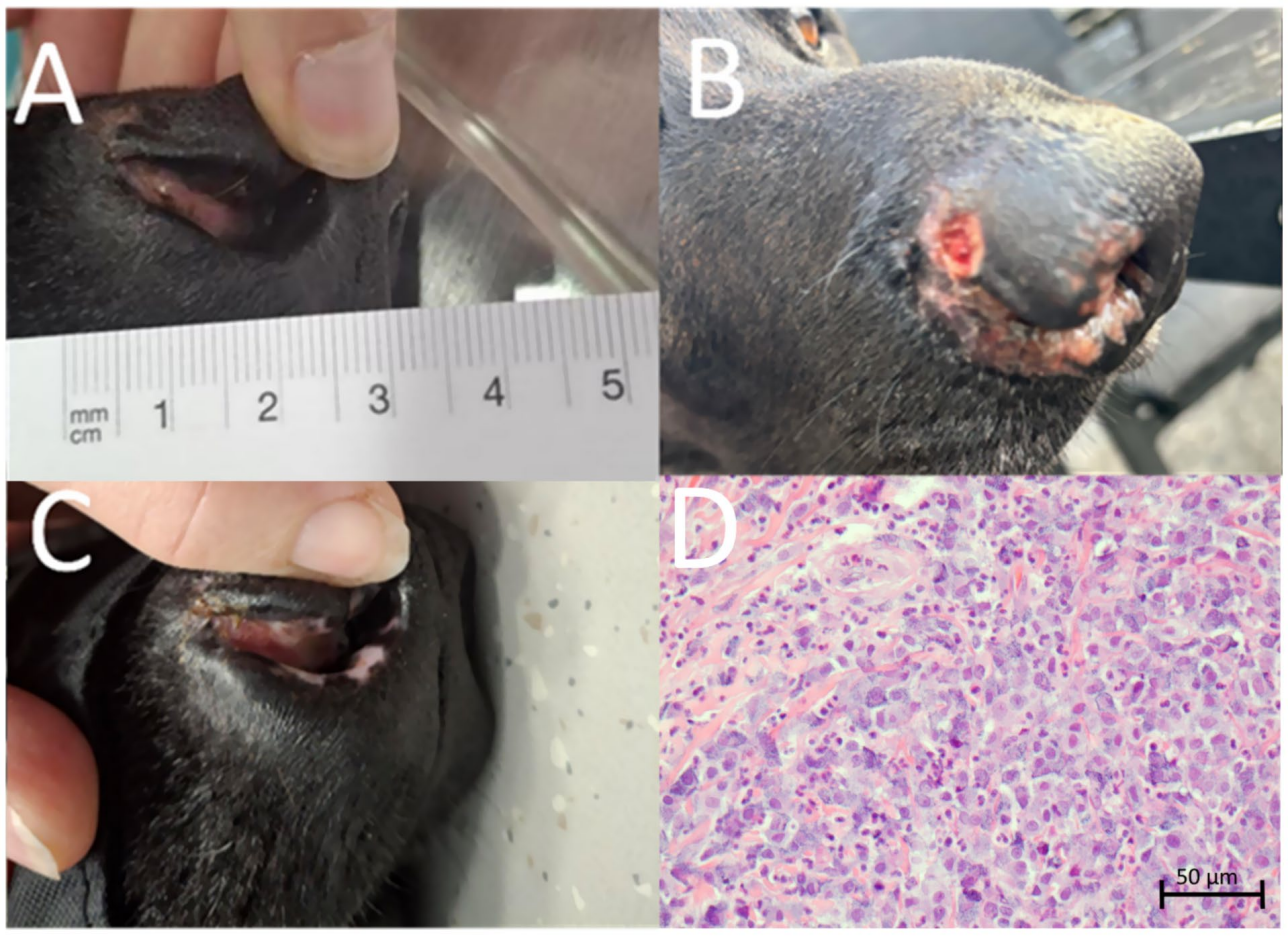
Timeline of the second treatment of the MCT on the nasal planum using ECT and IL-12 GET: at the time of treatment **(A)**, 1 week after treatment **(B)**, and 2 months after treatment **(C)**. Microscopic findings of the mast cell tumor that recurred 18 months after treatment: Sheets of tumor cells; hematoxylin and eosin **(D)**, 400×.

The dog remained free of clinical signs and visible disease until 18 months after treatment. However, when the tumor recurred, we performed a repeat treatment using the same combination of ECT and IL-12 GET. On this occasion, ECT was carried out with bleomycin (15,000/m^2^) using needle row electrodes, as the tumor appeared more on the outside of the nasal planum (Figure 5A). The tumor measured 1.2 × 1 × 0.8 cm in size. Two months after treatment, the tumor regressed, and after 6 months, the tumor was no longer visible (Figures 5B,C). In addition, a biopsy was performed during the second treatment. The microscopic examination confirmed an MCT, classified as Patnaik grade 2/Kiupel low grade (G2/LG). No mitosis was found in 10 high-power fields (HPFs) (2.37 mm^2^), and no invasion of blood or lymphatic vessels was observed (Figure 5D).

## Discussion

This case report describes a dog with an MCT treated using a multimodal approach combining ECT, IL-12 GET, and tyrosine kinase inhibitors. Histologically, the MCT was classified as Patnaik grade 2/Kiupel low grade based on morphological features, and the proliferation index (Ki-67) indicated low proliferative activity. However, the immunohistochemical analysis revealed a predominant KIT staining pattern II, a marker associated with shorter overall survival and a higher risk of local recurrence (24).

In accordance with the 2012 consensus recommendations (1), the dog was initially treated with tyrosine kinase inhibitors before starting the combined ECT + IL-12 GET therapy, which was initiated 1 month after the start of tyrosine kinase inhibitor treatment. ECT is a well-established treatment for canine MCTs, demonstrating high local efficacy (12). Its combination with IL-12 GET has been shown to extend disease-free and progression-free intervals (19). Similarly, in this case, we achieved durable local control for 18 months following treatment with the single-needle electrode. After a second treatment using a different electrode modality, local control was maintained for over 6 months, and the dog remains in remission at the time of writing the report. One of the challenges in applying ECT to tumors in anatomically complex or deep-seated sites (30, 31) lies in the geometry and rigidity of available electrodes. The available electrodes may have fixed geometries (linear or hexagonal) or variable configurations using single-needle electrodes, which have been applied in the treatment of deep-seated tumors such as hepatocellular carcinoma (32); however, in this approach, multiple needle insertions are required to generate a sufficiently strong and homogeneous electric field. Here, we report the successful use of a single-needle electrode for a tumor located in the nasal cavity—a site otherwise difficult to access. Previous studies have demonstrated the feasibility of using single-needle electrodes for nasal squamous cell carcinoma in dogs (33), achieving an electroporated field of 3 mm around a 6 mm electrode. In our case, however, we used an electrode with a diameter of 1.3 mm, which was demonstrated to generate an effective electric field extending up to 10 mm (22). Importantly, thinner electrodes that can produce a sufficient electric field also minimize trauma to the surrounding tissue.

In this case report, we also provide the first long-term observation of treatment efficacy using this type of electrode. The dog remained disease-free for 18 months before tumor relapse. The benefit of combining ECT with IL-12 GET in prolonging progression-free survival has been previously demonstrated (19), and in this case, the addition of tyrosine kinase inhibitors may have provided further benefit, given the c-kit expression profile. The multimodal approach, integrating local (ECT) and systemic (IL-12 GET and tyrosine inhibition) strategies, appears to offer superior outcomes in high-risk cases, such as mucocutaneous MCT with lymph node involvement, where reported median survival times range from 9 to 14 months (34).

In addition, histopathological analysis was performed at three time points: Before treatment and at 6 and 12 months after treatment. Six months post-treatment, the biopsy of the tumor site showed replacement of neoplastic tissue with mature connective tissue, consistent with findings reported by Salvadori et al. (21) in dogs treated with ECT and IL-12 GET. Similar to the study by Salvadori et al., we also observed reduced proliferative activity, decreased microvessel density, and changes in immune infiltrates. Specifically, the numbers of CD3+ and CD20+ cells were lower in the connective tissue replacing the tumor, showing dynamic changes in lymphocyte infiltration similar to those described by Salvadori et al., where CD3+ cells peaked at 4 weeks and declined by 8 weeks post-treatment, while CD20+ cells showed minimal changes (21).

In conclusion, this report highlights the feasibility and effectiveness of using a bipolar single-needle electrode for ECT in anatomically challenging, deep-seated tumors. The combination of tyrosine kinase inhibitors with ECT and IL-12 GET provided durable local tumor control; however, in this single case report, we cannot determine the individual contribution of each agent to the overall treatment effectiveness. In addition, after the patient experienced a relapse, we altered our treatment approach by changing both the cytostatic agent and the type of electrode used. This was because the tumor had become more visible, which allowed us to use standard needle electrodes, and the change in cytostatic was implemented due to the relapse. While these results are promising, larger clinical studies are needed to fully evaluate the efficacy of bipolar single-needle electrodes for the treatment of tumors in difficult anatomical locations using electroporation-based therapies.

## Data Availability

The original contributions presented in the study are included in the article/supplementary material, further inquiries can be directed to the corresponding authors.
